# Collectanea Medica: Consisting of Anecdotes, Facts, Extracts, Illustrations, &c.

**Published:** 1820-04

**Authors:** 


### COLLECTANEA MEDICA:

CONSISTING OF

### ANECDOTES, FACTS, EXTRACTS, ILLUSTRATIONS, &c.

***Relating to the History or the Art of Medicine, and the Auxiliary Sciences.***

Floriferis ut apes in saltibus omnia libant,
Omnia nos itidem depascimur aurea dicta.

### *Description of the Scutellaria Lateriflora.*

SCUTELLARIA LATERIFLORA, Pursh. G. 490. sp. 1. Didynamia, Gymnospermia.—*Nat. order*, Labiatæ. *Gen. ch.* Calyx ore integro, post florescentiam clauso operculato. Corollæ tubus elongatus. *Sp. ch.* Ramosissima, glabriuscula; foliis longissime petiolatis ovatis dentatis; caulinis subcordatis, racemis lateralibus foliosis.

The following popular description is subjoined, to enable ordinary herbalists to find it.

The scullcap grows in moist shady places, in most parts of America from Canada to Carolina: it is from one to three feet high, bearing small blue flowers, and blossoms from the latter end of July to September. It belongs to the same order of plants as the catmint, sage, mint, horehound, hyssop, &c. (the Labiatæ,) which are distinguished by the following characters: viz. the flowers two-lipped; the stamens four in number, two long and two short; the style single; the calyx persistant, containing four naked seeds; and the stalk quadrangular, with the branches and leaves opposite.

*Generic Characters of Scutellaria.*—Calyx or flower-cup small, terminated by two entire lips, which, after flowering, close and cover the seed with an orbicular lid; on the upper side of the tube of the calyx is a small process, which, from some fancied resemblance to a shield, has given name to the plant. After florescence, and as the seed advance to maturity, this enlarges and becomes a prominent part of the calyx. Corolla, two-tipped, the upper one compressed at its sides and arched: it is three-cleft, with the middle lobe much the largest; the lower lip broader than the upper one, and slightly notched; the tube of the corolla long, and somewhat bent at its lower extremity.

Pursh gives nine species of the American scullcap. The *lateriflora,* of which we are speaking, is distinguished from the others by the branched character of the herb, its oval, serrated, sharp-pointed, leaves, supported on long petioles or leaf-stalks; (this last character serves to distinguish it from the two species which most nearly resemble it, viz. the *nervosa* and the *galericulata*, which have the leaves sessile, or nearly so, attached to the stalk without a petiole;) the lower leaves are subcordate, or somewhat heart-shaped. The *fructification* (whence the specific name of the plant is derived) proceeds from the axilla, or base of each of the leaves of the branches. It is a raceme, of which the garden-currant is an example, and consists of a long peduncle, supporting, as on a common axis, the flowers ranged on it singly, by means of short equal pedicels. The whole plant is perfectly bare, void of any furze or down, of a light-green or glaucous colour, and in its general appearance not unlike the common mint (mentha sativa.) The stalk near the root is reddish; and the fibres of the root, on being washed, are of a pale-yellow.—[From Dr. Spalding's *History of its Use as a preventive of Hydrophobia.*]

---

### *Some Physiological Experiments and Reflections.* By Mr. Brodie.

[From Dr. Cooke's *Treatise on Nervous Diseases.*]

*Experiment* I.—Mr. Brodie divided in a dog the skin and muscles which lie before the axillary plexus of nerves, and afterwards the nerves themselves. He then divided the remaining skin and muscles, the cellular membrane, and every other part connecting the anterior extremity to the trunk, with the exception of the axillary artery and vein; so that the vessels were completely insulated, and formed the only connexion between the limb and the trunk. The divided edges of the skin were united by sutures. Twenty hours afterwards, an incision having been made in the fore-arm, the arteries bled freely, and the blood was of a bright-scarlet colour. The muscles, by means of the voltaic battery, were readily made to contract; and, when several pairs of plates were employed, the contractions of the muscles did not appear to be less powerful than those which arise from the stimulus of the will.

*Experiment* II.—Mr. Brodie removed the whole of the posterior part of the spinal marrow of a frog. The wound readily healed, but the hind legs became of course paralysed. Five months afterwards, the muscles of the hind legs were found still capable of powerful contractions under the influence of the voltaic battery. At the end of six months more, the muscles still retained their contractile power. The frog was then killed: the wound was found completely cicatrized; there was not the smallest appearance of regeneration of that portion of the spinal marrow which had been destroyed. From these experiments, Mr. Brodie thinks himself warranted in concluding that the second deduction of M. Le Gallois is not correct, viz. that the principle which animates each part of the body resides in that part of the spinal marrow from which the nerves of the part arise.

Mr. Brodie has also favoured me with the communication of some experiments formerly made by him, which strongly militate against the opinions of the celebrated Haller, respecting some important physiological points. As the experiments and the opinions of Mr. Brodie on this subject have not been made public, the account of them here introduced will probably be read with a considerable degree of interest.*

"Haller supposed," says Mr. Brodie, "and the opinion is still, I believe, generally received, that the immediate cause of the contractions of the heart, is the stimulus of the blood applied to the inner surface of its cavities; so that the circulating fluid is to the movements of this organ what volition, communicated by means of the nerves, is to the movements of the voluntary muscles. The following experiments have induced me to doubt the correctness of Haller's doctrine on this subject."

*Experiment* I.—"In a rabbit nearly full grown, I divided, at one incision, both the carotid arteries near their origin, and also the other vessels in the lower part of the neck. There was a profuse hemorrhage, and in thirty seconds all appearance of sensibility and voluntary motion had ceased. Two minutes after the division of the blood-vessels, I opened the chest, and I found the heart acting one hundred and forty times in a minute. The contractions were regular and vigorous. The muscular parietes were firm and unyielding to the touch, and the appearance of the heart did not at all differ from that in the living animal, except that it was of a smaller size, in consequence of its cavities not being distended with blood. An incision having been made in the right ventricle, a very small quantity of blood oozed from it; but the left ventricle was entirely empty."

*Experiment* II.—"A rabbit was inoculated with the Woorara poi-

* These experiments, as well as those which relate to the doctrines of Le Gallois, were read to the Royal Society in the year 1813, and were ordered to be printed; but the publication of them was postponed at the desire of Mr. Brodie, who wished for farther time, to enable him to complete the subject by additional experiments.

son; and, when apparently dead, the circulation was kept up by means of artificial respiration, as explained by me in the Philosophical Transactions for the years 1811, 1812. The thorax having been opened, the heart was found acting one hundred and forty times in a minute. I divided the aorta and vena cava inferior immediately below the heart. There was of course a profuse discharge of blood, and the heart became almost instantly empty; but this produced no alteration in its action. At the end of two minutes, the contractions were still as regular, frequent, and vigorous, as in the living animal. From this time the contractions became less frequent, and also feeble and irregular."

"In these experiments, (Mr. Brodie says,) the action of the heart continued apparently unaltered for at least two minutes after that viscus and the great blood-vessels were empty of blood; a circumstance which cannot well be explained, on the supposition that the contractions of the heart are produced by the stimulus of the blood in its cavities; since it is to be supposed, that the contractions of a muscle would cease immediately on the removal of the stimulus which produced them. It is well known, (Mr. Brodie observes,) that, if an animal be suffocated, the heart continues to act, circulating dark-coloured blood for a certain space of time after respiration has ceased;" but, in prosecuting this enquiry further, he found "that a *regular* and *vigorous* action of the heart continues for a *longer* period, when it is completely empty of blood, than when it is distended with blood of a dark colour."

"Is it not probable, (says Mr. Brodie,) that the immediate cause of the contractions of the heart may be certain impressions communicated through the nervous system, rather than the stimulus of the blood in its cavities? Do not many circumstances, besides the experiments just related, tend to show that this is a more just and satisfactory explanation of the circulation of the blood than that proposed by Haller?"

Mr. Brodie remarks, 1st. "That, if a person lose a considerable quantity of blood, the contractions of the heart are rendered more frequent; as if, by the more rapid circulation, to make up for the diminished quantity of the circulating fluid. Were the blood the stimulus which excites the heart to contract, the loss of blood ought to diminish the frequency of the contraction; since a longer time would be necessary for a sufficient quantity of the stimulus to be accumulated in any one of the cavities.

2d. "As the valves of the heart open in the direction of the circulation, the auricle and ventricle of each side, with respect to the blood entering the heart, may be considered as forming a single cavity, of which, if the blood were the stimulus producing the contraction, the whole should dilate and contract at the same instant, instead of the auricle and ventricle dilating and contracting in succession, as we find to be the case.

3dly. "The action of the heart is rendered more frequent and more powerful in one case; more slow and feeble, or perhaps altogether suspended, in another case, where the mind is agitated by rage, fear joy, or any other violent emotion. These circumstances are almost inexplicable on the supposition that the blood is the stimulus by which the action of the heart is excited; but they may be easily explained, on the supposition that the contractions of the heart depend upon certain impressions communicated through the nervous system.

4. "This last hypothesis is more conformable to what is observed in other organs. For example, respiration is produced by the never-ceasing action of the diaphragm, as the circulation of the blood is by that of the heart; and no one can doubt, that the diaphragm is stimulated to contract by impressions which it is constantly receiving through the medium of the phrenic nerves; for, if we divide these nerves, it ceases to contract. The uterus, in parturition, is excited to contract, not by the stimulus of its contents, but by the influence of the nervous system; as is proved by this circumstance, that, in cases of extra-uterine conception, when the uterus contains only the membrana decidua, it begins to contract at the same period of time as in common pregnancy, when it contains the fetus and other membranes."

---

### *Observations respecting the Gymnotes, and other Electric Fish.*

[From Baron Humboldt's *Historical Relation of his Voyage in America.**]

The Spaniards confound, under the name of *tembladores (tremblers)*, all electric fish. They exist in the sea of the Antillas, on the coasts of Cumana. The Indians of Guayqueries, who are the most skilful fishers and most industrious people of those regions, brought us a fish which, as they said, benumbed their hands. This fish rises up the little river of Manzanares. It was a new species of *raja*, the spots on the sides of which are but slightly visible, and which much resembles the *torpedo* of Galvani. The torpedoes, provided with an electric organ, which is visible externally from the diaphanous state of the skin, form a genus or sub-genus different from the rajas, properly speaking.† The torpedo of Cumana was very lively, and extremely vigorous in his movements, and yet the electric shocks he gave us were but very weak. They became stronger on galvanising the animal by the contact of zinc and gold. Other *tembladores*, real gymnotes, or electric eels, inhabit the Rio Colorado, the Guarapiche, and many rivulets which traverse the Chaymas Indies. They enter also the great rivers of America,—the Oroonoko, the Amazon, and the Meta; but the force of the current and depth of the water in those

* In his last voyage in America, Baron Humboldt made many original observations respecting the gymnotus and torpedo, which are highly interesting to the physiologist expressly, as well as to the naturalist in general; and, as the history of the Voyage of this philosopher is, from its great price, perhaps not in the possession of many of our readers, we shall give some extracts from it, relating to the animals above designated.—Edit.

† Cuvier, *Regne Animal*, t. ii. p. 156. There are in the Mediterranean, according to Risso, four species of electric torpedoes, which were formerly confounded under the name of *raja torpedo:* namely, *torpedo narke, torpedo unimaculata, torpedo galvani,* and *torpedo marmorata.* The torpedo of the Cape of Good Hope, on which Mr. Todd has recently made some experiments, is undoubtedly a nondescript species.

parts prevent the Indians from catching them. They see these fish less frequently than they feel electric shocks from them, in swimming or bathing in the river. It is in the Llanos, especially in the environs of Calabozo, between Morichal and Aniba and Abaxo, that the ponds of stagnant water and the overflowings of the Oronooko (the Rio Guarico, the Canos of the Rastro, Berito, and Paloma), are filled with gymnotes. We wished at first to make our experiments in the house we inhabited at Calabozo; but the fear of the electric shocks of the gymnotus is so great, and their effects so much exaggerated by the people, that for three days we could not procure any of these animals, although they may be caught with facility, and we had promised the Indians two piastres for each large and vigorous fish they would bring us. This fear of the Indians is the more extraordinary, because they do not resort to a means for preventing the shocks, in which, they state, they have much confidence. They tell white men, when they are interrogated respecting the effects of the *tembladores*, that they may be touched with impunity, if we chew tobacco. This fable of the influence of tobacco on the electric powers of these animals, is as general on the continent of South America, as the belief of sailors is in the effect of garlic and suet on the magnetic needle.

Impatient of this long delay, and obtaining only very indeterminate results from our observations on a living gymnotus very much weakened, which they had brought us, we went to Cano de Bera, to make our experiments in the open air on the side of the water. We sat out on the 19th of March early in the morning, for the little village called Rastro de Abaxo; whence the Indians led us to a small river, which, in seasons of great drought, forms a pool of muddy water, surrounded by beautiful trees,* clusia, amyris, and mimoses with odoriferous flowers. The catching of the gymnotes with a net is very difficult, because of the extreme agility of these fish. We would not employ the *barbasco*, that is to say, the roots of the *piscidia erithryna*, the *jacquinia armillaris*, and some species of *phyllanthus*, which, thrown into a pond, intoxicate or stupify the fish: this measure would have weakened the gymnotes. The Indians told us, they would *fish with horses*.† We could not imagine what this extraordinary way of fishing could be; but we soon saw our guides return from a savannah, where they had collected several wild horses and mules, which they drove before them into the pool. The noise made by the splashing of the horses made the fish rise from the bottom of the water, and prepare for the combat. These yellowish and livid eels, similar to large water-snakes, were then seen swimming on the surface of the water, and pressing themselves against the bellies of the horses and mules. A battle between animals so different presented a very picturesque scene. The Indians, armed with harpoons and long sharp canes, closely surrounded the pool; some of them, too, climbed up

---

* Amyris *lateriflora*, A. *coriacea*, Laurus *pichurin*, Myroxilon *secundum*, Malpighia *reticulata*.

† *Embarbascas con cavallos*: properly, make the fish *intoxicated or sleepy* by means of horses.

the trees whose branches hung over the water, and, by their cries and weapons, prevented the horses from escaping from the water. The eels defended themselves by repeated discharges of their electric batteries. For a long time they seemed likely to become victorious. Many horses sunk under the violence of the shocks they received over all the organs most essential to life; benumbed by the force and frequency of them, they disappeared under the water. Others, panting for breath, with erect mane, haggard eyes, and signs expressive of extreme anguish, rose again, and endeavoured to escape from the storm around them; but the Indians drove them back again into the middle of the water. Some, however, escaped the vigilance of the fishermen, and gained the shore, stumbling at every pace, and stretching themselves on the sands, apparently benumbed by the electric shocks of the gymnotes. In less than five minutes, two horses were drowned. The eel, being five feet in length, pressing itself along the belly of the horse, makes a discharge from the whole extent of his electric organ, and thus attacks at once the heart, the coeliac plexus, and the whole of the abdominal nerves. It is obvious that the effect in the horse must be greater than what it is in a man, when he only touches the animal by one of his extremities. The horses are, probably, not killed, but merely benumbed, and are drowned, from their inability to raise themselves during the combat between the rest with the gymnotes. We at first had no doubt but that the battle would end with the death of all the horses engaged in it; but the impetuosity of it gradually diminished, and the gymnotes, fatigued, dispersed. They have occasion for a long repose and an abundance of nutrition, to repair what they have lost of their galvanic power.* They timidly approached the margin of the pool, when they were taken by means of little harpoons attached to long cords. When the cords were perfectly dry, the Indians drew them up without receiving any shocks. In a few minutes we had obtained five large gymnotes, most of which were but slightly wounded. Several others were taken towards the evening by the same means. The temperature of the water in which the gymnotes usually live, is about 26° or 27° Reaumur, (90° or 92° Fahrenheit.) They assured us that their electric power diminished in colder water; and it is very remarkable, as a celebrated naturalist has observed, that animals, generally, endowed with organs productive of electric powers, the effects of which are sensible to man, are not found in the air, but in an electro-conductive fluid. The gymnotus is the largest of the electric fish. I have seen some of them which were from five feet to five feet three inches in length. The Indians declared that they had seen still longer. We found that a fish which was three feet ten inches long weighed twelve pounds. The transverse diameter of the body was, without including the anal fin, three inches and five lines, (very nearly four inches English measure.)

The gymnotes of Cano de Bera are of a fine olive-green colour. The part below the head is yellow, mingled with red. Two rows of

* The Indians assured us, that, when a troop of horses were driven into that pool two days in succession, no horse is killed on the second day.

small yellow spots are placed symmetrically along the back, from the head to the point of the tail: each spot surrounds an excretory opening; and the skin of the animal is constantly covered with a mucous matter, which, as VOLTA proved, conducts electricity twenty or thirty times more powerfully than pure water. It is very remarkable, that no electric fish hitherto discovered is provided with scales.

The gymnotus, like the common eel, likes to respire the air on the surface of the water. The latter is known to creep during the night on the grassy banks of rivers; but we must not conclude, with M. BAJON, that the fish would die if it were not to come and respire air without the water. I have seen a very vigorous gymnotus die on the dry ground, after having darted out of the adjacent pool. M. PROVENCAL and myself have proved, by our researches on the respiration of fishes, that their gills, when wet, are capable of performing the functions, both of decomposing atmospheric air, and of abstracting the oxygen dissolved in water. They do not suspend their respiration in the air, but absorb gaseous oxygen, like a reptile provided with lungs. It is well known that carps are fattened by feeding them out of water, taking care to moisten from time to time their gills with some wet moss, to prevent them from becoming dry. Fish open their bronchial valves more in oxygen gas than in water; but their temperature is not raised, and they live as long in vital air as in a mixture of 90 parts of azote and 16 of oxygen. We have found that tenches, placed under bell-glasses filled with air, absorb in an hour half a cubic centimetre (about one-sixth of a cubic inch English measure) of oxygen. This action takes place in the gills alone; for, fish having cork-collars adapted to them, and placed thus with their head outwards and the rest of the body in a vessel filled with air, do not absorb the oxygen by the rest of the body. It appears, that respiration of air is effected by the medium of an extremely thin layer of water over the surface of the gills.

The swimming-bladder of the gymnotus is two feet five inches long in a fish three feet ten inches in length. It is separated from the skin by a mass of fat, and is situate on the electric organs, which fill more than two-thirds of the animal. The same vessels that are insinuated between the laminæ of these organs, give also numerous branches to the exterior surface of the bladder just mentioned. One hundred parts of the fluid contained in this bladder, I found to be constituted of 96 parts azote and 4 oxygen.

The medullary substance of the brain presents but a slight analogy with the albuminous and gelatinous matter of the electric organs, but these two substances have this in common,—they receive a large quantity of arterial blood, which is deprived of its oxygen in them.*

* We stop, at present, at the conclusion of these preliminary observations, because we wish to give an account of Baron Humboldt's experiments on the electric powers of the gymnotes in an uninterrupted manner, and the limits of the Journal will not permit us to do this in the present Number.—EDIT.

